# Unravelling the impact of fat content on the microbial dynamics and spatial distribution of foodborne bacteria in tri-phasic viscoelastic 3D models

**DOI:** 10.1038/s41598-023-48968-8

**Published:** 2023-12-09

**Authors:** Lisa Purk, Melina Kitsiou, Christina Ioannou, Hani El Kadri, Katherine M. Costello, Jorge Gutierrez Merino, Oleksiy Klymenko, Eirini G. Velliou

**Affiliations:** 1 Bioprocess and Biochemical Engineering Group (BioProChem), Department of Chemical and Process Engineering, University of Surrey, Guildford, GU2 7XH UK; 2 Centre for 3D Models of Health and Disease, Division of Surgery and Interventional Science, University College London, Charles Bell House, 43-45 Foley Street, Fitzrovia, London, W1W 7TY UK; 3 School of Biosciences and Medicine, University of Surrey, Guildford, GU2 7XH UK

**Keywords:** Microbiology, Applied microbiology, Bacteria, Biofilms

## Abstract

The aim of the current study is to develop and characterise novel complex multi-phase in vitro 3D models, for advanced microbiological studies. More specifically, we enriched our previously developed bi-phasic polysaccharide (Xanthan Gum)/protein (Whey Protein) 3D model with a fat phase (Sunflower Oil) at various concentrations, i.e., 10%, 20%, 40% and 60% (v/v), for better mimicry of the structural and biochemical composition of real food products. Rheological, textural, and physicochemical analysis as well as advanced microscopy imaging (including spatial mapping of the fat droplet distribution) of the new tri-phasic 3D models revealed their similarity to industrial food products (especially cheese products). Furthermore, microbial growth experiments of foodborne bacteria, i.e., *Listeria monocytogenes*, *Escherichia coli*, *Pseudomonas aeruginosa* and *Lactococcus lactis* on the surface of the 3D models revealed very interesting results, regarding the growth dynamics and distribution of cells at colony level. More specifically, the size of the colonies formed on the surface of the 3D models, increased substantially for increasing fat concentrations, especially in mid- and late-exponential growth phases. Furthermore, colonies formed in proximity to fat were substantially larger as compared to the ones that were located far from the fat phase of the models. In terms of growth location, the majority of colonies were located on the protein/polysaccharide phase of the 3D models. All those differences at microscopic level, that can directly affect the bacterial response to decontamination treatments, were not captured by the macroscopic kinetics (growth dynamics), which were unaffected from changes in fat concentration. Our findings demonstrate the importance of developing structurally and biochemically complex 3D in vitro models (for closer proximity to industrial products), as well as the necessity of conducting multi-level microbial analyses, to better understand and predict the bacterial behaviour in relation to their biochemical and structural environment. Such studies in advanced 3D environments can assist a better/more accurate design of industrial antimicrobial processes, ultimately, improving food safety.

## Introduction

The latest estimations in 2022 by the WHO report 600 million incidences and 420,000 deaths each year globally due to foodborne illnesses, which is predicted to increase^1^. A major contributor to these foodborne illnesses is bacterial contaminations of food products. At the same time, the food industry is moving towards alternative treatments to classical sterilisation approaches. This trend is driven on the one hand by the increasing consumers’ demand for minimally processed food products with milder treatments and, on the other hand due to the global need for more sustainable and environmentally friendly industrial processing approaches^2–12^. Therefore, achieving microbiologically safe food products is a major and evolving challenge, especially as the efficacy of a treatment is hugely affected by the structural and physico-chemical characteristics of each food product. Those characteristics can influence the growth pattern of potential microbial contaminants, consequently affecting the efficiency of a treatment in inactivating them^3,7,8,12–20^. Furthermore, the efficiency of an antimicrobial treatment can be affected by intra-species interactions in cases of cross-contamination of a food product with multiple pathogens as well as in products where there is a rich background microbiota^8,12,21–25^.

According to the ‘European Union One Health 2020 Zoonoses Report’ by the European Food Safety Authority (EFSA), common bacterial pathogens found in a variety of food products are *Listeria monocytogenes*, *Escherichia coli* and *Pseudomonas aeruginosa*^26^.

*Listeria monocytogenes (L. monocytogenes),* causes listeriosis and has the highest death rate among common foodborne associated pathogens, especially affecting pregnant women, the elderly and vulnerable population^24^. According to Endrikat et al*.*, around 90% of human listeriosis outbreaks are associated with the consumption of ready-to-eat (RTE) foods^27^. The European Centre for Disease Prevention and Control (ECDC) reports for 2021 high level of listeriosis cases, especially for Germany, France and Italy^28^. Due to the long incubation period of up to 3 months, listeriosis is hard to recognise and difficult for an epidemiological investigation, leading to outbreaks lasting for several years, as it was the case in South Africa from January 2017 to July 2018, with 1060 confirmed cases and a mortality rate of 27%^29^. Additionally, *L. monocytogenes* is a highly competitive, stress resistant and biofilm forming bacterium, resulting in high persistence in its environment and better resistance to preservative methods and cleaning agents^30–33^.

*Escherichia coli (E. coli)* is another common foodborne pathogen, which is harmful for humans and has led frequently to food-associated outbreaks all over the world^34–36^. It is mostly transmitted through unhygienic practices, where even the low infection dose makes the toxin producing bacterium particularly dangerous, as regular outbreaks around the world demonstrate^26,35–37^. A recent outbreak was reported in 2019 in Scotland (UK) in association with contaminated RTE sandwiches resulted in 39 cases of Shiga toxin-producing *E. coli* (STEC)^38^. Similarly to *L. monocytogenes*, also *E. coli* is biofilm forming, which makes this contaminant a dangerous enemy for the food industry^39,40^.

The same applies for the pathogenic *Pseudomonas aeruginosa (P. aeruginosa)*, which is known for the biofilm formation potential and high production of extracellular polymeric substances (EPS). High levels of EPS are further associated with the formation of a protective layer around pathogenic bacteria and therefore reduced efficiency of preservation technologies or cleaning techniques^23,41,42^. Although *P. aeruginosa* is commonly associated with the spoilage of meat and dairy products^43–45^, it has been also associated with severe outbreaks; as it was reported in 2018 in the USA with 20 cases linked to antimicrobial resistances (AMR)^46^.

Further to pathogens, there are also bacteria which are used in foods for beneficial reasons. Commonly, those are lactic acid bacteria (LAB) or yeasts. Their application is widely spread in various foods, especially in dairy and meat products^47–49^. Therefore, this study also includes a LAB strain, as representative for beneficial bacteria in foods. *Lactococcus lactis* (*L. lactis*) is a commonly used beneficial bacterium and mainly used in fermentation processes, e.g., in the dairy industry to produce yogurt. The metabolised lactic acid acts as a preservative of those food products, along with the production of the bacteriocin nisin^50^.

In the last decades, the majority of research on foodborne bacterial dynamics (growth and inactivation) has mostly been conducted (i) in actual food products such as cheeses, sausages, vegetables or fruit juices^51–56^, or (ii) in liquid broths where bacteria grow planktonically (see examples Cornu, Kalmokoff and Flandrois, Valdramidis et al*.,* and Velliou et al*.*)^57–60^. However, in the case of food products, considering the high complexity of most foods along with batch-to-batch variation the results are mostly specific to the product, making it difficult to extrapolate a general trend and/or predict the microbial behaviour in other systems. Furthermore, in the case of liquid broths, they are not representative of the actual features of a food product. More specifically, although the structures and composition of some food products can be very simple, most foods are complex solid or solid(like) three-dimensional (3D) structures^17,61^. In such solid or solid(like) 3D structures bacteria form clusters, colonies and/or biofilms and consequently their dynamics, environmental and/or treatment responses vary hugely from simple liquid systems. Therefore, conducting in vitro research in solid or solid(like) 3D in vitro models is crucial, to obtain a fundamental understanding of the bacterial dynamics and/or treatment response in well-defined environments which mimic closely real food systems^6–8,12,19,60,62–64^.

Most solid or solid(like) in vitro models in literature are simple single (mono-)phase models usually consisting of liquid broth media, enriched with a gelling/solidifying agent, e.g., agar, xanthan-gum, gelatin, k-carrageenan^16,18,60,65–67^. All those studies highlight differences in the bacterial dynamics as a result of growth or inactivation in a solid environment, as compared to simple liquid broths. The changes of bacterial behaviour can further result in reduced efficiency of both traditional and novel food safety control measurements. For example, Velliou et al. and Noriega et al. showed that *E. coli*, *Listeria* and *Salmonella* were much more resistant to heat inactivation (at lethal temperatures) when grown and subsequently (heat) treated in a solid xanthan based system, compared to liquid broth^3,18^. Generally, when bacteria grow in colonies they are progressively exposed to increased acid conditions as a result of their metabolism, which can make them more resistant to other environmental stresses via the cross-protection mechanism^3,60,68–70^. Costello et al. also reported that the type of bacterial growth, i.e., planktonic vs colony(like) in a Xanthan Gum-based monophasic model, affected the efficiency of Cold Atmospheric Plasma and nisin against *Listeria*, i.e., the antibacterial effect was reduced when bacteria grew in aggregates/colonies^9^.

More recently, there have been some studies in/on food models with increased structural and/or biochemical complexity. Bacterial cell distribution and/or clustering has been reported to alter in more complex model systems. For example, Boons et al*.* developed a biphasic gelatin/dextran model in which *E. coli* was observed to grow selectively in the dextran phase^71^. Verheyen et al. developed fat rich tri-phasic systems, i.e., a viscous system containing Xanthan Gum, an emulsion, an aqueous gel, and a gelled emulsion, and compared the growth of *L. monocytogenes* on the surface of those complex systems^14,72^. In terms of microbial kinetics, the growth of *L. monocytogenes* was higher on the viscous system as compared to the gelled system. Furthermore, the growth of *L. monocytogenes* was faster (shorter lag phase and higher microbial growth rate) in presence of fat at low temperatures (4 °C). In terms of spatial distribution, for increasing fat concentration, the growth on the fat–water interface was more dominant as compared to the aqueous face, information that could not be obtained with macroscopic kinetics.

Within our group, we recently developed a novel bi-phasic system consisting of a polysaccharide phase (Xanthan Gum) and a protein phase (Whey Protein). After studying the growth and spatial distribution of *Listeria innocua* (*L. innocua*) and *L. lactis* on/in this bi-phasic system we observed no substantial differences in the growth kinetics as compared to a mono-phasic system, but we observed selectivity in the spatial growth of the microorganisms at the early stages of growth. More specifically in mono-cultures, micro-colonies formed at the early stages of growth, were mostly located on the protein phase of the models, that pattern chanced in co-cultures of *L. innocua* and *L. lactis*^7,8^.

*The above studies highlight that there is need for further research in 3D model systems of increased structural and biochemical complexity, in order to improve our understanding of the impact of complex structure on the microbial growth and response to treatment approaches.*

To that end, the aim of the current study was to develop novel multi-phasic 3D model systems and to study the macroscopic and microscopic growth of foodborne bacteria on those systems. More specifically, building on our previously developed bi-phasic (protein/polysaccharide) model^7^ we introduced a third phase, i.e., fat in the model. Different concentrations of fat were used to mimic the fat content of various food products. Rheological, structural, and physicochemical characterisation of the models took place. Thereafter, the microbial evolution of *L. monocytogenes*, *E. coli, P. aeruginosa* and *L. lactis* was studied on the developed 3D models for different temperatures. More specifically, macroscopic kinetics were obtained in combination with advanced microscopy, which enabled observations of the bacterial spatial spatiotemporal organisation/distribution in the 3D models at colony level.

## Material and methods

### Preparation of tri-phasic viscoelastic food model systems

As previously mentioned, novel tri-phasic fat rich models (emulsions) were developed, building on our recent bi-phasic Xanthan Gum/Whey protein model^7,8^. The novel food models consisted of 10% (w/v) Whey Protein Powder (WPI, Prolacta® 95 Instant Native Whey Protein Isolate, Bacarel, UK), 5% (w/v) Xanthan Gum (XG, Xantural®75; CP Kelco, UK), 3% (w/v) tryptone soya broth (TSB, Oxoid Ltd., UK), 1% (w/v) sodium chloride (NaCl), 0.6% (w/v) yeast extract (YE, Oxoid Ltd., UK) and different concentrations of oil. More specifically, commercially available, food grade sunflower oil (Sainsbury’s Sunflower oil, UK) was added in the mixture at various concentrations, i.e., of 10%, 20%, 40% and 60% (v/v), to mimic fat rich food products^52,73–75^. For the mixing, initially WPI, TSB, NaCl and YE were dissolved in deionised H_2_O followed by addition of the oil followed by homogenisation, using a laboratory homogeniser (Omni International Inc., USA) at 2000 rpm for 2 min. Thereafter, the XG was added in the mixture and further homogenisation took place for 5 min at 2000 rpm. The developed model systems were autoclaved (30 min, at 121 °C), to achieve sterile conditions.

### Characterisation of tri-phasic viscoelastic food model systems

#### Rheological characterisation

The rheological behaviour of the developed viscoelastic models was examined at 7 °C, 25 °C and 37 °C, by conducting dynamic oscillatory measurements, as previously described by Costello et al.^7^. Those temperatures were selected in order to represent relevant temperatures during food processing, distribution, and storage. The storage modulus G΄ (Pa), the loss modulus G′′ (Pa) and the loss tangent tanδ = G′/G′′ were measured as functions of angular frequency ω from 0.1 to 100 (rad/s). G′ and G′′ represent the elasticity and viscosity of the model, respectively. The loss tangent (tanδ = G′/G′′) determines whether a sample can be characterised as viscous fluid (tanδ > 1) or elastic solid (tanδ < 1). The G′, G′′ and tanδ values were obtained using a Discovery Hybrid (HR-2) rheometer (TA Instruments, USA), with a parallel plate geometry (40 mm diameter, 0 ° angle, S/N 113475).

#### Textural characterisation

Texture characterisation of all developed food model systems took place at 7 °C, 25 °C and 37 °C, using a Texture analyser with accompanying Texture Exponent 32 software (TA.XT plus, Stable Micro Systems, Surrey, UK). More specifically, all tri-phasic model samples as well as bi-phasic fat-free control samples were placed on a plastic container (diameter 50 mm) which was then stored in a constant temperature chamber (set at 7 °C, 25 °C or 37 °C) and allowed to equilibrate to the set temperature. Thereafter, the samples were compressed under a 30 mm cylindrical aluminium probe (P/50) at a trigger force of 1 G and a test speed of 1 mm/sec. Two compression cycles at 50% of the initial sample height (4 mm) were applied using a pre-test speed of 1 mm/sec and a post-test speed of 4 mm/sec. Experimental data obtained from the force–time curves were used to determine adhesiveness, cohesiveness, gumminess, and hardness. Data analysis was performed using Origin® 2018 (OriginLab Corporation).

#### Physico-chemical characterisation

The pH values, the moisture content (w_m_) and the water activity (a_w_) of the food models were measured.

The pH values of the models were determined using a pH meter (Edge pH Meter HI2020, USA). Measurements were conducted in triplicate (for three independent replicate models) and measured at four different locations within each sample tube, at 25 °C, to ensure capturing of any potential gradients in the pH values. There were no significant variations within each sample, confirming the good homogenisation of the model components.

The determination of the moisture content was performed according to IDF 80-1:2001 (E), ISO 3727-1:2001 (E). More specifically, the samples (5 g) were transferred to pre-weighted aluminium oven dishes and heated in a drying oven at 102 °C for 2 h. Thereafter, the samples were transferred to a desiccator for 15 min for cooling prior to weighting. The moisture content was determined by the following equation:

1 $${W}_{m} \left(\%\right)= \frac{\left({m}_{1}-{m}_{0}\right)-({m}_{2}-{m}_{0})}{({m}_{2}-{m}_{0})}*100\%$$

where *w*_*m*_ is the moisture content of the sample (%), *m*_*0*_ is the mass (g) of the dish, *m*_*1*_ is the mass (g) of the sample before drying and *m*_*2*_ the mass (g) of the sample after drying.

The water activity (a_w_) was measured using the HygroLab C1 Bench-Top Indicator (Rotronic AG, Switzerland). The food model samples with the different fat contents were spread into a sample container, fitting the sample holder of the device. The probe was placed on the sample and the reading was captured. The device recorded the water activity value, as well as the temperature to ensure consistency and comparability of the data.

### Structural microscopic analysis of the tri-phasic viscoelastic food model systems

#### Determination of the oil droplet size in the 3D tri-phasic models

The distribution of the oil droplets within the models as well as the determination of their size took place with light microscopy (Swift, UK, at 10× and 40× magnification), for all fat concentrations under study (10%, 20%, 40%, 60% v/v). For each model approximately 500 oil droplets were measured and at least three samples were analysed. Data analysis was performed using ImageJ software (Java-based program, National Institute of Health, USA).

#### Confocal laser scanning microscopy of the 3D tri-phasic models

For a more in-depth spatial analysis of the distribution of the different components of the models and their structure, confocal laser scanning microscopy (CLSM) was employed, for all 3D models under study. More specifically, sections of the models were stained with 30 µL of the red dye Rhodamine B (0.01% v/v), which is selective to protein and with 30 µL of the green dye BodiPy (0.1% v/v), which is selective to fat. Thereafter, samples were incubated for 15 min in the dark and washed three times with 1 mL deionised H_2_O. The CLSM (Ti-Eclipse Inverted Microscope System, Nikon Instruments Europe) z-stack imaging was carried out by using a 10× magnification. At least three different samples per condition were studied and at least 10 images per sample were obtained, to ensure reproducibility of the visual observations.

#### Confocal laser scanning microscopy of real food products

To structurally compare the developed fat-rich tri-phasic models with real food products, especially with respect to the spatial distribution of the fat phase, several fat rich products were chosen, stained and visualised using CLSM (as described for the models in section "Determination of the oil droplet size in the 3D tri-phasic models"). More specifically, the following food products were analysed (if not mentioned otherwise, the products are the UK Supermarket Sainsbury’s own brand): (1) Pork liver paté (30% w/w fat content), (2) Coarse pork sausage (19% w/w fat content), (3) Smooth pork sausage (18% w/w fat content, Richmond Sausages, UK), (4) Low fat Philadelphia cheese (2.5% w/w fat content), (5) Philadelphia cheese (11% w/w fat content), (6) Camembert cheese (20% w/w fat content, Le Roustique, France), (7) Mascarpone cheese (42% w/w fat content).

#### Scanning electron microscopy

To structurally analyse the developed models at higher resolution, scanning electron microscopy was employed, as previously described^8^. Similar moulds as per CLSM were used. The surface area was the same as for all other experiments (2.3 cm^2^). However, the samples were only few millimetres thin to enable faster/better dehydration prior to imaging. The food models were placed in 24 well plates and fixed via incubation at room temperature for 1 h in 3% formaldehyde solution (made with Dulbecco’s Phosphate Buffered Saline (DPBS) in a 1:1 ratio). After the formaldehyde was removed, the samples were serially dehydrated in 10%, 20%, 40%, 60%, 80% and 100% (v/v) ethanol (99.6%) at room temperature for 10 min per each ethanol concentration and washed twice with DPBS, modified without CaCl and MgCl_2_ (D8537, Sigma-Aldrich Ltd., UK) for 5 min. After the last washing, the samples were left overnight in the fume hood with the lid ajar. Thereafter, the dehydrated, fixed samples were mounted on a 13 mm aluminium stub (Agar Scientific) and sputter coated with gold twice to a thickness of 6 nm using an Emitech K550X Sputter Coater (Quorum Technologies, Ashford, UK) with a target current of 20 mA for 1 min. Then, the samples were observed under a JEOL JSM-7100F SEM microscope operated at 5 kV. At least three different samples per condition were studies and at least 10 images per sample were obtained, to ensure reproducibility of the visual observations.

### Statistical analysis

For statistical analysis the results obtained in the sections "Rheological characterisation" and "Textural characterisation" were analysed for significant differences with a two-sided ANOVA test (*p* = 0.5), followed by a TUKEY test. The results of the sections "Physico-chemical characterisation" and "Determination of the oil droplet size in the 3D tri-phasic models" were analysed for significant differences with a one-sided ANOVA test (*p* = 0.5), followed by a TUKEY test.

### Bacterial strains and inoculum preparation

The bacteria used in this study were *L. monocytogenes* EDG^−e^^76^, *E. coli* MG1655, *P. aeruginosa* ATCC 15442 and *L. lactis* NZ9700. The bacterial stock cultures were stored at − 80 °C in a 70:30 TSBYE/Glycerol (50%, Sigma-Aldrich Ltd.,, UK) mixture and were cultured as previously described^3,7–9,12,60^, to reach stationary phase. More specifically, a loopful of thawed stock culture was inoculated in 15 mL of TSBYE growth medium for *L. monocytogenes*, *E. coli* and *P. aeruginosa* and in 15 mL of MRS growth medium for *L. lactis*. After 9.5 h at 37 °C (*L. monocytogenes*, *E. coli P. aeruginosa*) and at 30 °C (*L. lactis*) the cultures were sub-cultured by transferring 20 μL to 15 mL fresh medium and were cultured for another 15 h, until early stationary growth phase was reached. For growth kinetics (section “Macroscopic microbial kinetics on the surface of tri-phasic viscoelastic food model systems”) non-GFP (green fluorescence protein) strains were used, while for the microscopy imaging (section “Microscopic analysis of the microbial spatial distribution on the surface of tri-phasic viscoelastic food model systems”) the equivalent strains were used with the genetic modification to express GFP.

### Macroscopic microbial kinetics on the surface of tri-phasic viscoelastic food model systems

The early stationary phase cultures obtained as described above (section "Bacterial strains and inoculum preparation"), were appropriately diluted in phosphate buffered saline (PBS, Oxoid Ltd., UK) to a concentration 10^5^ CFU/mL. Thereafter, 33 µL of that microbial concentration was spread on the surface, resulting in a final cell concentration of 3.3*10^3^ CFU/disc (on a surface area of approximately 2.3 cm^2^). Model systems with 0%, 20% and 60% (v/v) were initially chosen to start with the lowest, the middle and the highest fat concentration. The samples were incubated for 24 h at 37 °C (optimal growth temperature for *L. monocytogenes*, *E. coli, P. aeruginosa*) and at 30 °C (optimal growth temperature for *L. lactis*). Samples were taken at frequent and appropriate time points, to obtain the microbial growth curves (hourly from time 0 h to 9 h and then at 12 h, 15 h and 24 h post-inoculation).

Subsequently, the samples were processed as previously described^7,8^. The samples were mixed with PBS on a 1/10 dilution and were then processed in a stomacher bag (BA6040, Seward, UK) for at least 1 min, to ensure good homogenisation (Colworth Stomacher 80, Seward, UK). Thereafter, 100 μL of sample was taken from the centre of the bag and followed by an appropriate serial dilution prior to plating on TSAYE/MRSA plates. The plates were then incubated at 37 °C (*L. monocytogenes*, *E. coli P. aeruginosa*) and at 30 °C (*L. lactis*) for 24 h. Plate counts were converted in CFU/mL. Experiment was conducted with at least 3 technical replicates and 3 biological replicates per time point to ensure consistency of growth kinetics.

### Mathematical modelling of the macroscopic microbial kinetics

To predict the microbial growth parameters, the explicit version of the Baranyi and Roberts model was fitted to the experimental data^77^:

2 $$lnN\left(t\right)=ln{N}_{0}+ {\mu }_{max}A\left(t\right)-\mathrm{ln}\left(1+ \frac{{e}^{{\mu }_{{max}^{\cdot A(t)}-1}}}{{e}^{\left({N}_{max}- {N}_{0}\right)}}\right)$$

with:

3 $$A\left(t\right)=t+ \frac{1}{{\mu }_{max}}\mathrm{ln}\left(\frac{{e}^{{-\mu }_{{max}^{\cdot t}}}+ \frac{1}{{e}^{\left(\lambda \cdot {\mu }_{max}\right)}-1}}{1+ \frac{1}{{e}^{\left(\lambda \cdot {\mu }_{max}\right)-1}}}\right)$$

where *N(t)* [CFU/mL] is the cell density at time *t*; *N*_*max*_ [CFU/mL] is the maximum value for *N(*t), that is, cell density at stationary phase; *μ*_*max*_ (1/h) is the maximum specific growth rate, and λ (h) is the lag phase duration. The MATLAB Version R2018b (MathWorks Inc., Natick, MA) was used for growth curve generation and data analysis. The mathematical model was fitted to each replicate data set with optimization of the fit achieved using the function lsqnonlin, which solves nonlinear least-squares problems, to minimize the sum of the squared errors. Standard deviations (SD) of the model parameter estimates were obtained via the variance–covariance matrix for each repeat. Independent experiments were performed with three biological replicates, the presented plots are an average fit for the combined replicates while the separate values of all independent experiments shown in the Appendix, Table S2.

### Microscopic analysis of the microbial spatial distribution on the surface of tri-phasic viscoelastic food model systems

To gain information on the spatial distribution of the bacterial cells at colony level within the developed viscoelastic models, further microscopic analysis was performed. More specifically, for the microscopic growth analysis, 3D models with fat concentrations of 0%, 20% and 60% (v/v) (chosen as described for section “Macroscopic microbial kinetics on the surface of tri-phasic viscoelastic food model systems”) were formed into discs with the same surface area as described before (see section “Macroscopic microbial kinetics on the surface of tri-phasic viscoelastic food model systems”) and stained with 0.1% of the red dye NileRed (fat specific dye) for 10 min. After washing, the 3D model samples were air dried prior to inoculation with 3.3*10^3^ CFU/disc (same inoculation level as the macroscopic growth experiments described above) of *E. coli*, *P. aeruginosa* and *L. lactis*, labelled with a GFP expression plasmid.

The inoculated samples were incubated in the dark at 37 °C (*E. coli*, *P. aeruginosa*) and at 30 °C for *L. lactis* and microscopically analysed with CLSM as described previously (see section "Confocal laser scanning microscopy of the 3D tri-phasic models"). More specifically, *E. coli* and *P. aeruginosa* were analysed at the lag phase (3 h), early-exponential phase (5 h), late-exponential phase (7 h) and stationary phase (24 h). For *L. lactis* the earliest GFP signals could be detected at the late-exponential phase (12 h) and stationary phase (24 h). This was due to the slower growth rate of *L. lactis* and also due to the chromosomal GFP modification for this strain (rather than plasmid) which both result to a later GFP expression.

## Results and discussion

### Tri-phasic viscoelastic food model systems

**Figure 1:**
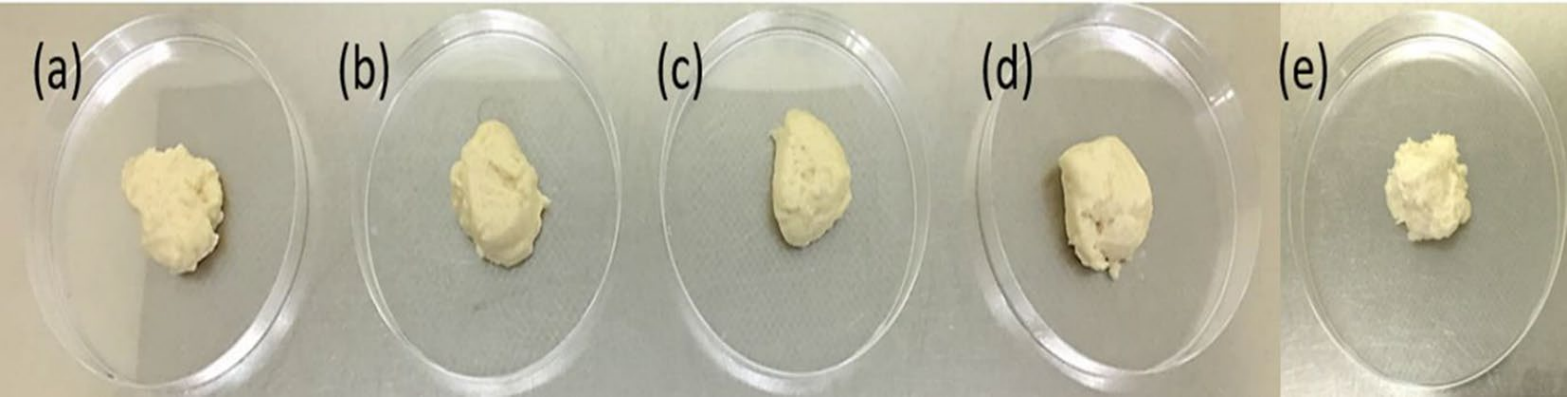
3D models under study: (**a**) bi-phasic (xanthan gum/whey protein) 3D model and (**b**–**e**) tri-phasic systems with xanthan gum/whey protein/fat viscoelastic 3D models of increasing fat content from left to right (10%, 20%, 40% and 60%).

As many food products contain fat, the addition of fat enables the development of even more realistic models and allows the possibility of studying the role of fat in (i) the development of the structure of the model and (ii) the growth and spatial distribution of bacteria within the models. Figure 1 shows four triphasic models with fat at concentrations of 10%, 20%, 40%, 60% (v/v), mimicking high lipid content food products^52,73–75^.

*To the best of our knowledge, this is the first time that such methodical approach has been used to study the impact of high fat concentrations on the bacterial evolution in tri-phasic 3D models.*

Our results are divided into two main parts: (i) characterisation of the 3D tri-phasic models (section "Characterisation of the 3D viscoelastic food models") and (ii) bacterial growth experiments on the surface of the 3D tri-phasic models (section "Multilevel (macro- and micro-) analysis of the spatiotemporal growth of foodborne bacteria in the novel tri-phasic viscoelastic 3D models").

### Characterisation of the 3D viscoelastic food models

#### Rheological characterisation

**Figure 2:**
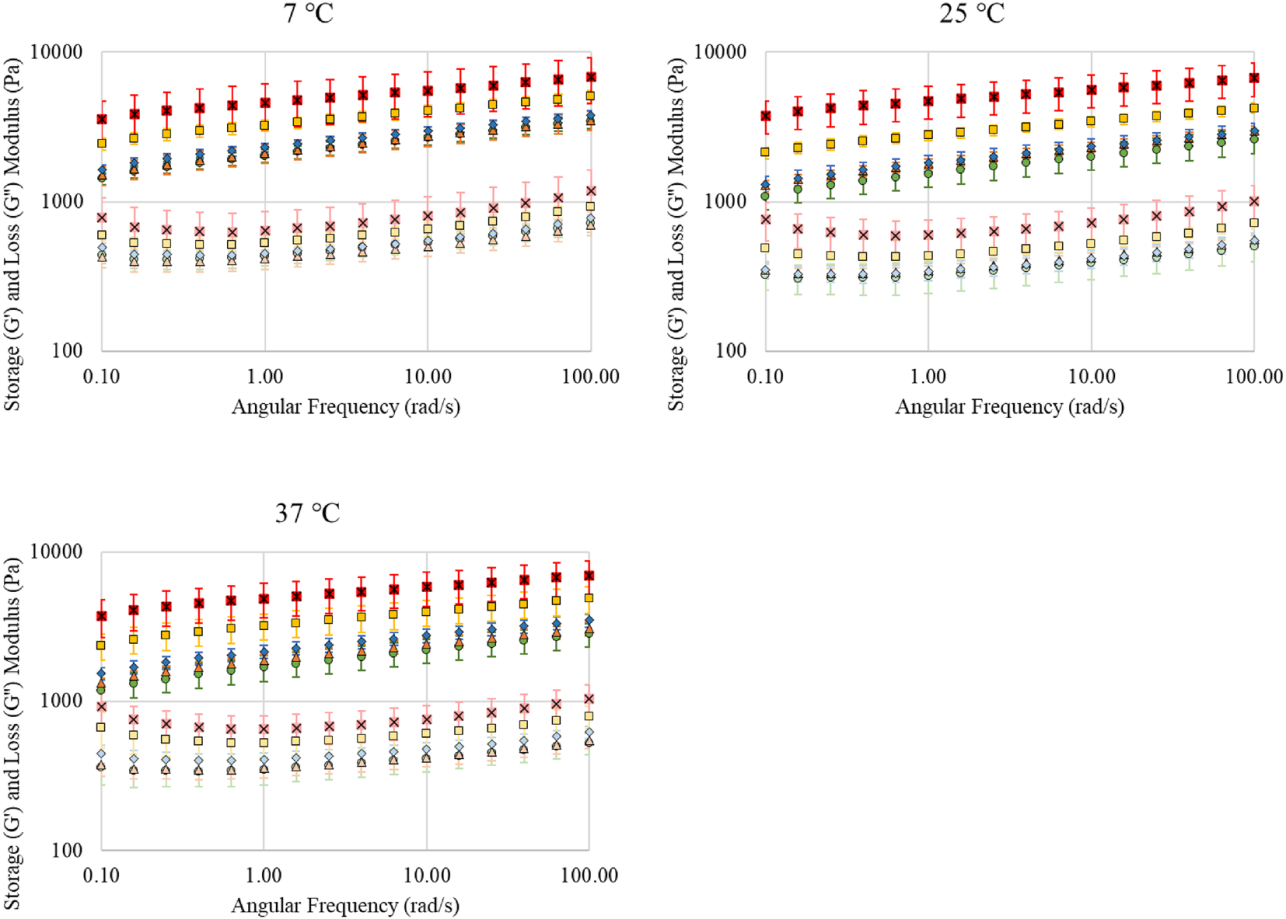
Rheological characterisation of the viscoelastic 3D models. The storage modulus G′ and the loss modulus G′′ (Pa) as a function of the angular frequency (rad/s) at 7 °C, 25 °C, and 37 °C. Where 0% fat for G′ is () and for G′′ (), 10% fat for G′ is () and for G′′ (), 20% fat for G′ is () and for G′′ (), 40% fat for G′ is () and for G′′ () and 60% fat for G′ is () and for G’’ ().

Rheological characterisation of all models under study was conducted as described in section "Rheological characterisation". The rheological parameters of the developed 3D models are illustrated in Fig. 2 and their values are also presented on Table S1 (of Appendix). As can be seen in Fig. 2 and Table S1 of Appendix, all models are viscoelastic.

The value of the storage modulus, *G′*, is much larger than the value of the loss modulus, *G′′* (with a loss tangent *tanδ* < 1 for all gels/models), indicating that elasticity dominates the flow. Similarly to our previously reported bi-phasic models^7,8^, for all novel tri-phasic gels no statistical differences of the values of the rheological properties were observed for changing temperatures, for the temperature range under study (7 °C, 25 °C, and 37 °C). Statistically significant differences were observed in the values of the storage modulus, *G′*, and loss modulus, *G′′*, for models of different fat concentrations (*p* < 0.05). More specifically, the model with the highest fat content (60%) had the largest storage modulus and loss modulus, as compared to all other models, indicating the highest viscoelasticity, i.e., stronger/stiffer, more firm gel, followed by the 40% fat model. No statistically significant differences were observed for lower fat, i.e., 20%, 10% and no fat 3D models, indicating their rheological similarity (Figure S2, Table S1 of Appendix).

Our tri-phasic 3D models are novel (in terms of their chemical composition) and therefore a direct comparison with literature is not possible, however, previous studies on food products or emulsions/gels show similarities with respect to the reported rheological properties^78^. For example Lucey, Munro and Singh reported increasing elasticity (G*′*) and stiffness (G*′′*) with increasing fat concentrations for yoghurt-like milk gels^79^. A similar trend was observed by Peressini et al. for mayonnaise emulsions and by Tunick and Guinee & O’Callaghan for different cheese products^80–82^. Chung et al*.* also reported that the elasticity (storage modulus) and viscosity (loss modulus) of biphasic semi-solid starch-based systems, consisting fat droplets (varying from 0.5 to 30%), increased with increasing fat content^83^. A study by Verheyen, Baka, et al. also showed increased elasticity and viscosity in an emulsified oil/water gel as compared to an aqueous gel^14^.

Overall, in our gels, the fat content is a major contributor affecting the viscoelasticity (strength of the gel/ stiffness), for high fat concentrations, i.e., 40% and 60% fat.

#### Textural characterisation

Textural characterisation, i.e., measurement of hardness, cohesiveness, gumminess, and adhesiveness of all developed viscoelastic models was conducted. Those textural properties and the results of the structural characterisation are summarized in Table 1.

**Table 1:** Textural characterisation of the developed viscoelastic food models.

Viscoelastic 3D models (fat concentration) (%)	Temperature (°C)	Hardness (g)	Adhesiveness (g*s)	Cohesiveness	Gumminess (g)
0	7	423.27 ± 14.74	− 458.85 ± 41.02	0.79 ± 0.02	333.80 ± 17.35
	25	396.13 ± 27.02	− 440.93 ± 44.62	0.82 ± 0.04	325.11 ± 36.88
	37	460.73 ± 73.82	− 470.34 ± 89.53	0.79 ± 0.03	366.78 ± 70.61
10	7	508.87 ± 37.42	− 505.10 ± 22.32	0.79 ± 0.03	397.15 ± 15.48
	25	402.47 ± 45.25	− 420.35 ± 46.20	0.81 ± 0.04	324.92 ± 30.36
	37	447.53 ± 40.13	− 462.19 ± 40.53	0.80 ± 0.03	358.11 ± 32.26
20	7	457.13 ± 62.60	− 464.02 ± 55.74	0.81 ± 0.06	363.52 ± 32.59
	25	451.13 ± 17.09	− 481.77 ± 11.56	0.81 ± 0.01	366.88 ± 16.92
	37	442.30 ± 25.03	− 500.46 ± 5.17	0.82 ± 0.03	361.11 ± 10.55
40	7	572.80 ± 49.29	− 515.60 ± 48.57	0.78 ± 0.04	445.18 ± 36.44
	25	482.33 ± 51.55	− 423.28 ± 33.34	0.82 ± 0.03	390.15 ± 25.11
	37	502.60 ± 91.28	− 404.56 ± 50.32	0.79 ± 0.07	384.31 ± 41.92
60	7	625.07 ± 46.98	− 133.92 ± 22.17	0.54 ± 0.02	337.22 ± 20.99
	25	535.23 ± 20.55	− 80.59 ± 19.58	0.49 ± 0.03	262.46 ± 3.49
	37	454.37 ± 49.13	− 95.53 ± 19.18	0.56 ± 0.03	254.24 ± 27.06

Mean values (± SD are presented) for hardness (g), adhesiveness (g*s), cohesiveness, and gumminess (g), for all viscoelastic models and temperatures under study.

Table 1 shows similar values for hardness, adhesiveness, cohesiveness, and gumminess for the model systems with 0%, 10%, 20% and 40% fat, for all temperatures under study. However, the 60% model system showed significant differences in the values of adhesiveness, cohesiveness, and gumminess as compared to the rest of our models. However, for the 60% model system only gumminess and hardness were affected by the temperature, i.e., showing a decreasing trend with increasing temperature.

Similar trends to our findings have been reported in literature. For example, Ningtyas et al*.* reports a decrease in textural parameters with increasing fat concentrations in cream cheeses (0.5–11.6% fat w/w)^75^. Similarly, Theophilou and Wilbey reported a decrease in hardness with increasing fat concentration (32–53% fat w/w) in halloumi cheeses^84^. Brighenti et al. observed a decrease in hardness for Neufchâtel cheese as well as fat-free cream cheese at temperatures above 25 °C^52^.

#### Physico-chemical characterisation

The pH, the moisture content and the water activity of the developed models were measured at room temperature and their values are summarised on Table 2.

**Table 2:** pH, Moisture content (W_m_) and Water activity (a_w_) for all developed 3D models under study (10%, 20%, 40% and 60%).

Model systems (fat concentration)		pH	Moisture content (W_m_) %	Water activity (a_w_) %	
(a)	**0%**	6.88 ± 0.07^c,d,e^	71.163 ± 6.32^b,c,d,e^	0.951 ± 0.005	^–^
(b)	**10%**	6.74 ± 0.10^d,e^	58.890 ± 4.62^a,c,d,e^	0.950 ± 0.005	^–^
(c)	**20%**	6.40 ± 0.01^a,e^	46.854 ± 5.03^a,b,d,e^	0.956 ± 0.005	^–^
(d)	**40%**	6.31 ± 0.01^a,b^	29.062 ± 1.41^a,b,c^	0.954 ± 0.005	^–^
(e)	**60%**	6.25 ± 0.01^a,b,c^	21.973 ± 2.94^a,b,c^	0.955 ± 0.005	^–^

Statically significant different relations are indicated by the corresponding letters or – for no differences. The significance was determined with *p* = 0.5.

Generally, there are significant, but small differences in the pH values of the models. More specifically, a slight decrease of the pH value was observed with increasing fat concentration in the 3D models and a more drastic decrease in moisture content, whereas the water activity remains stable (Table 2).

Similarly to our findings, Sánchez-Macías et al. also reported that the pH decreased for higher fat cheese products as compared to low-fat cheese^85^. Generally, all our 3D food models show similar pH values with processed cheese products^13,82,86^. Further, all bacteria under study are neutrophiles (range pH 5.5–8.5). Therefore, the differences in the pH values between the model systems should not have a great effect on the bacterial growth kinetics. With respect to the moisture content (W_m_), as shown in Table 2, it significantly decreases with increasing fat content. The trends we report are in agreement with Lee et al., who observed that an increased moisture content resulted in an increase of the pH value in cheese spreads^87^. Similarly, McCarthy et al., observed that the moisture content and the pH value of cheese products decreased with increasing fat content^73^. The water activity (a_w_) of our 3D models was not affected by changes in the fat concentration. This is expected, i.e., as the a_w_ is the ratio of partial pressure of water vapour in the product (p) to that in presence of pure water p_(o)_, and the water ratio is the same for all the 3D models. The a_w_ value of our food models is representative of various foods, e.g. mozzarella cheese or liver paté^88^. In conclusion, we do not expect that the above physico-chemical characteristics would affect majorly the bacterial kinetics in our models. Nevertheless, in general, they are important characteristics that should be considered when interpreting changes in growth kinetics.

#### Structural microscopic analysis of the tri-phasic viscoelastic food model systems

##### Determination of the oil droplet size

**Figure 3:**
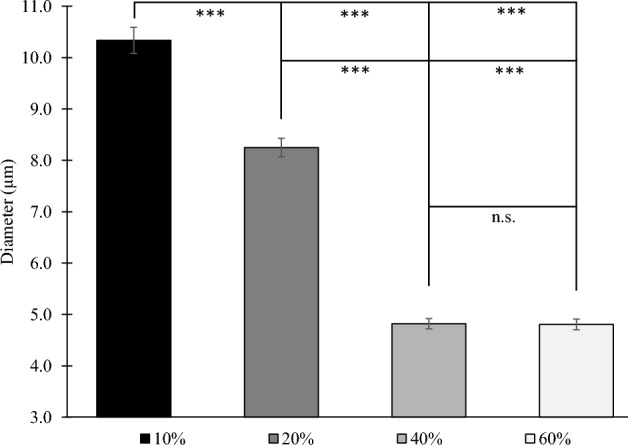
Average oil droplet size, as determined by light microscopy (×10 magnification) for all 3D food models under study (10%, 20%, 40% and 60%). Significance levels are indicated by braces and asterisks in the graphs (***p = 0.001 | n.s. = no significant difference).

The oil droplet size of the tri-phasic food model samples was determined by ImageJ software (particle size counting), of images of the 3D models, obtained by light microscopy (see Figure S1 of Appendix). The average fat droplet size decreased as the fat concentration increased, i.e., from 10.335 ± 0.253 to 4.805 ± 0.106 μm, with no statistical differences between 40 and 60% fat (Fig. 3). The correlation of fat droplet size and fat concentration is also reported in other studies for food products, like various dairy and meat (paté) products^89–93^, as well as for oil-in-water emulsions^94^. With respect to previous studies on in vitro models a similar trend to our findings was reported by Verheyen, Baka et al. in a fish-based model with fat concentrations of 5%, 10% and 20%^95^.

##### Confocal laser scanning microscopy of the tri-phasic 3D models

**Figure 4:**
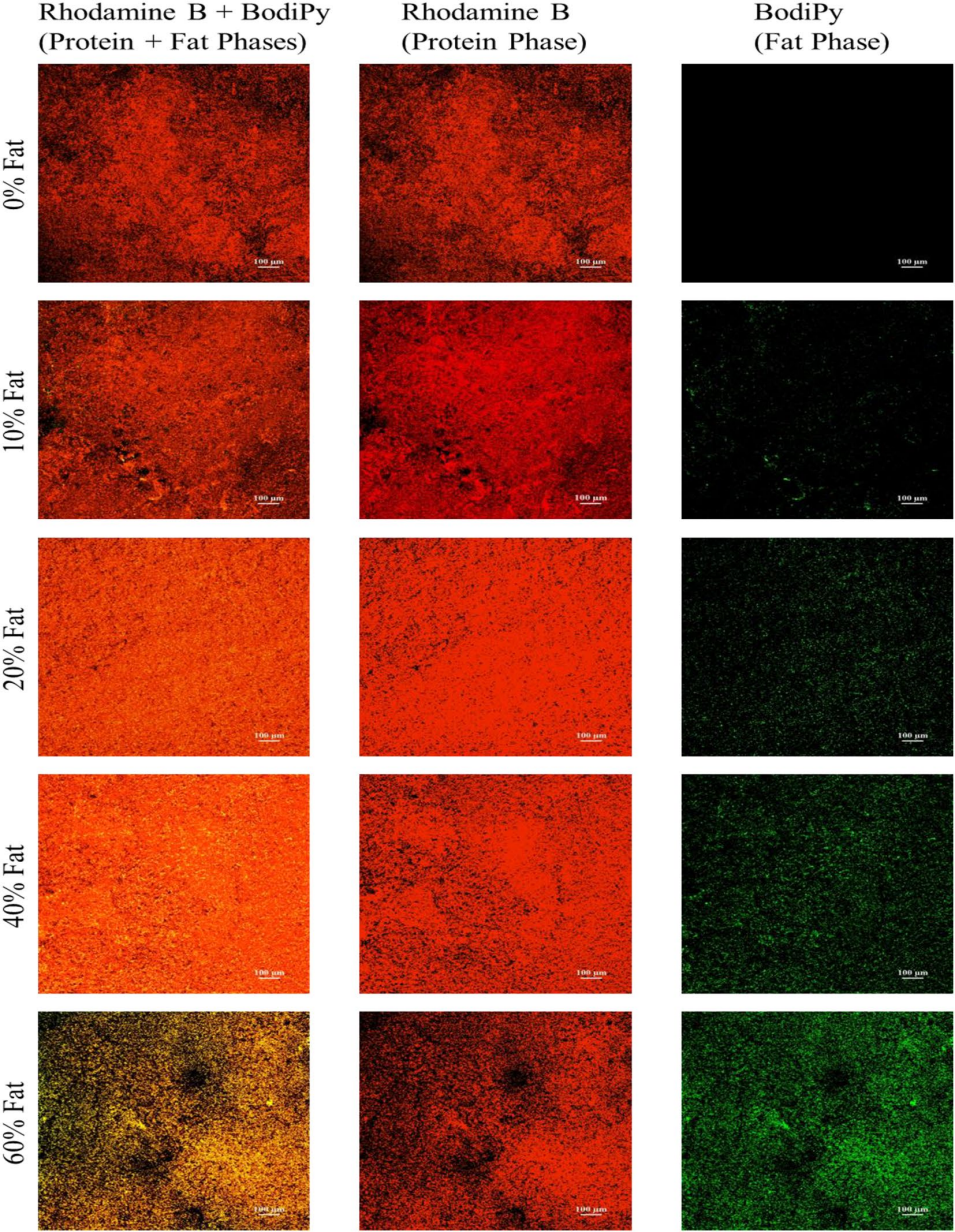
CLSM images of the surface of the developed food model systems: (**a**) 0% fat, (**b**) 10% fat, (**c**) 20% fat, (**d**) 40% fat and (**e**) 60% fat. The samples were stained in red with 0.01% Rhodamine B (protein phase) and in green with 0.1% BodiPy (fat phase). All z-stack images were taken with a 10× magnification.

As previously mentioned, appropriate selective staining of the protein and fat phases of the developed viscoelastic models took place, followed by CLSM analysis of sections of the model. Such analysis enabled the visualisation of the different phases of the models as well as their distribution (Fig. 4). As can be seen in Fig. 4, the fat is homogeneously distributed in the 3D models for all fat concentrations under study. Furthermore, the fat droplet size is decreased with increasing fat concentrations, confirming the light microscopy observations. Similar distribution and shape of fat globules have been reported for fat rich food products or models in literature. For example, Ørskov et al. reported similar fat droplet distribution to our models in cheese lab models (consisting of starch, casein and palm oil with 24% fat)^96^. Similar CLSM images have been also reported by Abhyankar, Mulvihill and Auty^97^ for milk gels containing whey protein powder and oil concentrations of 0–15% (w/w).

**Figure 5:**
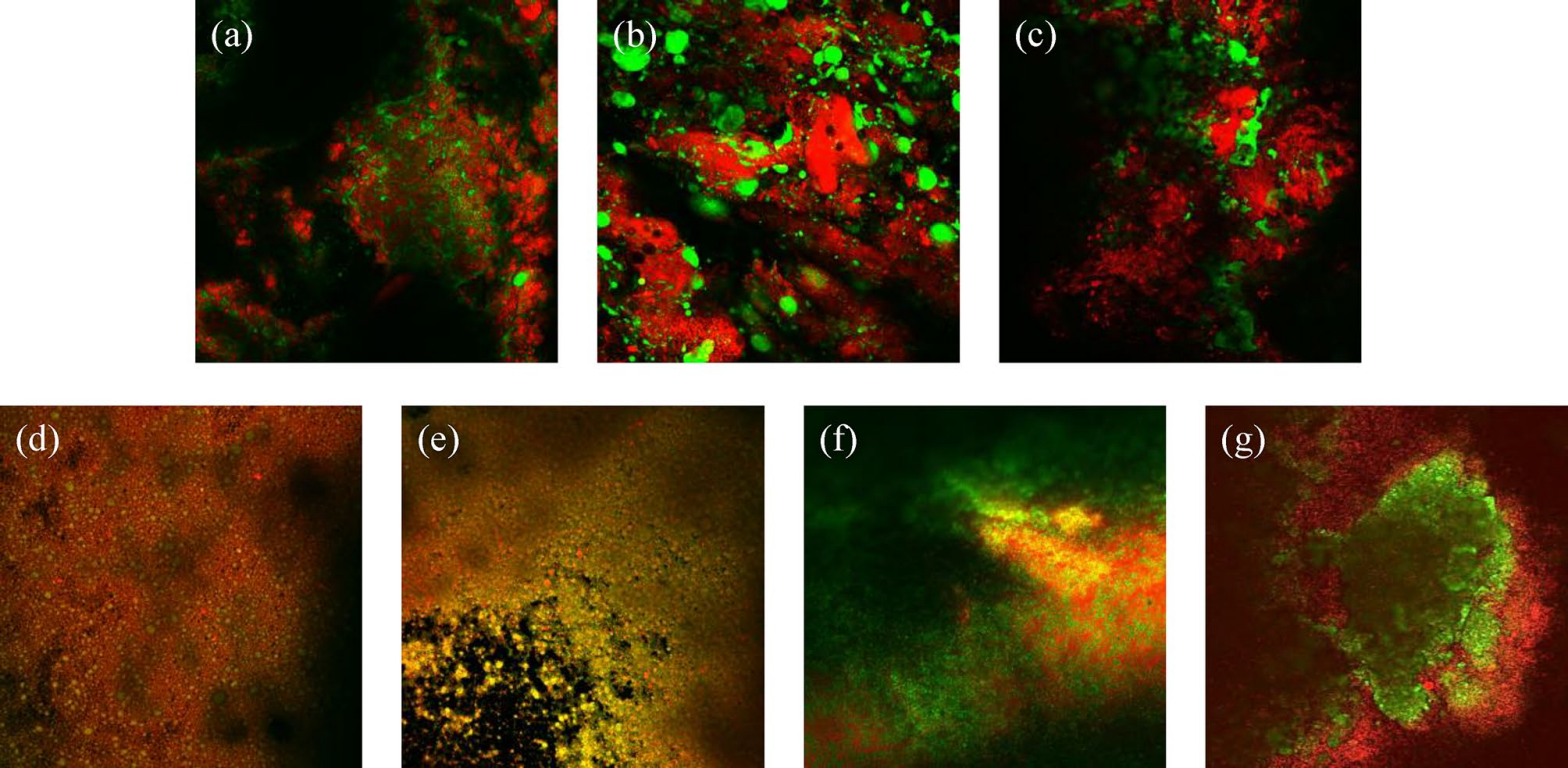
CLSM images of meat products: (**a**) liver paté (30% fat), (**b**) sausage (19% fat) and (**c**) smooth sausage (18% fat), and of dairy products: (**d**) low fat Philadelphia (2.5% fat), (**e**) Philadelphia (3.5% fat), (**f**) camembert (20% fat) and (**g**) mascarpone (42% fat). All samples were stained with 0.01% Rhodamine B (protein phase) and in green with 0.1% BodiPy (fat phase). All z-stack images were taken with a 10× magnification.

Considering the fact that our 3D models are novel, and their composition is unique, we Considering the fact that our 3D models are novel, and their composition is unique, we generated CLSM images of real food products, to enable a direct comparison to the developed 3D viscoelastic models. Specifically, cheese products and meat products with fat concentrations similar to our models were analysed (Fig. 5). Our CLSM images are similar to other studies, for similar food products. For example, CLSM images of different meat products reported by Huang et al. and meat model systems by Liu and Lanier showed very similar fat globules and distributions to the ones we report for liver pate and sausage Fig. 5a,b^98,99^. Furthermore, similarities between our obtained CLSM images for the cheese products analysed (Fig. 5d–g) have been reported in other studies^89,91,100^.

As can be seen in Fig. 5, the fat distribution and the fat globule size are highly product- specific, and they can vary even for the same fat concentration between different food products. For example, as can be seen in Fig. 5, the fat distribution and fat droplet size are very different between smooth sausage (Fig. 5c) and camembert cheese (Fig. 5f), despite the similarity in the actual fat concentration, i.e., 18% to 20% (w/w) respectively. Such variation between food products has also been reported in other studies. For example, a study by Auty reported that meat products have bulkier fat globules than cheese products (as analysed by CLSM)^101^.

When comparing our food models (Fig. 4) to real food products (Fig. 5), the fat distribution of the models is very similar to the one of cheese products, highlighting that our developed models are good surrogates of cheese.

##### Scanning electron microscopy (SEM) of the tri-phasic 3D models

**Figure 6:**
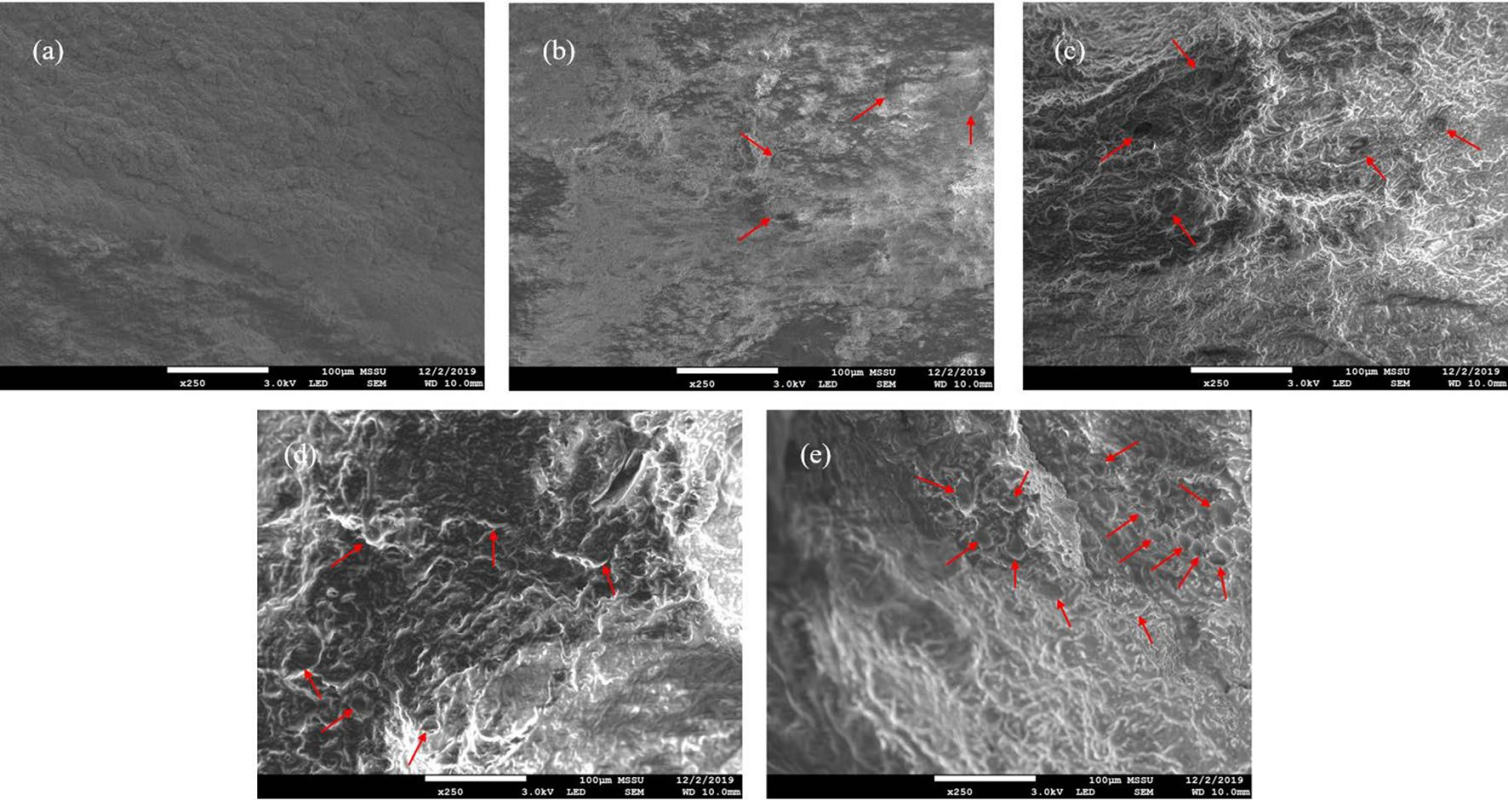
Scanning electron microscopy images of the surface of the 3D models for all fat concentrations under study: (**a**) 0% fat, (**b**) 10% fat, (**c**) 20% fat, (**d**) 40% fat and (**e**) 60% fat. Red arrows indicating examples for fat globule craters. All images were taken with 250 × magnification.

For a visualisation of the structure of the developed models at higher resolution, scanning electron microscopy (SEM) was employed. As can be seen in Fig. 6, there is very clear structural difference between the bi-phasic (0% fat) food model and the fat rich tri-phasic 3D models, with the latter appearing more complex and highly pitted, whereas the 0% fat food model appears to be much smoother and has no visible craters. The surface of the models containing fat show craters of perfectly round shapes and more sharp edges (highlighted by red arrows in Fig. 6), which are the locations of the fat droplets. Similar SEM images of low-fat cheddar cheese and cheddar cheese were shown by Hickey et al. and Romeih et al., as well in a study of various cheese products conducted by Ong et al.^100,102,103^. Furthermore, in alignment with our CLSM images (Fig. 4) as well as our light microscopy observations (Figure S1 of Appendix), the quantity of these craters in the SEM images (fat globules) increased in number and decreased in size, with increasing fat concentration. The SEM images also revealed that the surface topography of the developed food models is naturally uneven, rough, and irregular.

*Overall, the structural, textural, and microscopic analysis of the developed food model systems highlighted that they are comparable to real food products (especially cheese products), indicating their suitability, as food-surrogates, for fundamental microbiological studies.*

### Multilevel (macro- and micro-) analysis of the spatiotemporal growth of foodborne bacteria in the novel tri-phasic viscoelastic 3D models

As previously mentioned, several food related bacteria were inoculated on the surface of viscoelastic 3D models for 0%, 20% and 60% fat and incubated at their respective optimal growth temperatures for 24 h. Specifically, *L. monocytogenes*, *E. coli* and *P. aeruginosa* were incubated at 37 °C and *L. lactis* at 30 °C. Thereafter (i) the macroscopic surface growth kinetic data were obtained and mathematically modelled (section "Macroscopic surface growth dependency of foodborne bacteria on the fat concentration of viscoelastic food model systems" below) and (ii) the microscopic spatial distribution on the surface of the complex tri-phasic viscoelastic 3D models was imaged and analysed (section "Microscopic analysis of microbial spatial distribution on the tri-phasic 3D models" below).

#### Macroscopic surface growth dependency of foodborne bacteria on the fat concentration of viscoelastic food model systems

**Figure 7:**
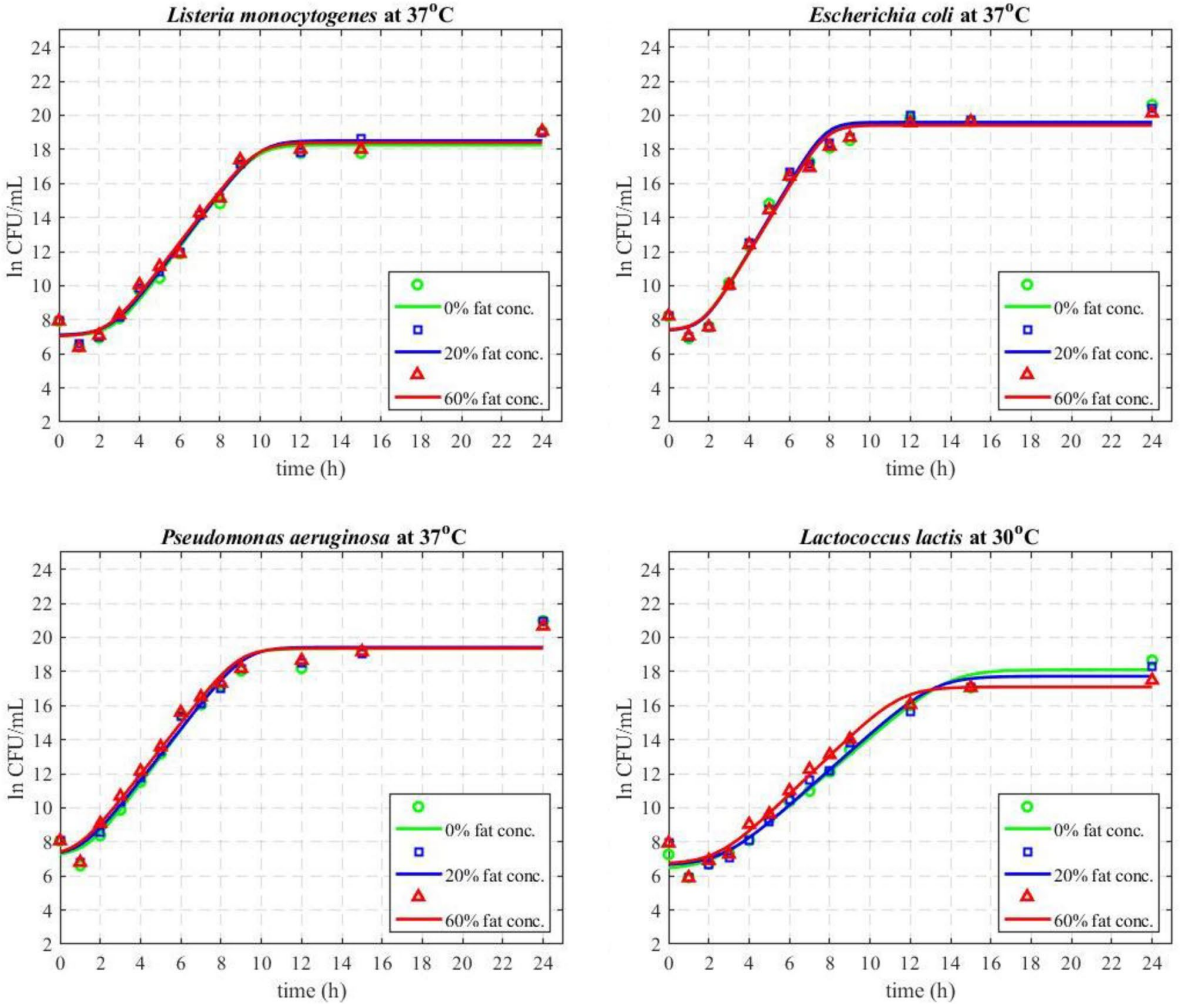
Fit of the Baranyi and Roberts mathematical model on the experimental data (macroscopic surface growth kinetics) of *L. monocytogenes*, *E. coli* and *P. aeruginosa* at 37 °C and *L. lactis* at 30 °C.

Figure 7 shows the fit of the Baranyi and Roberts growth model to the microbial growth mean experimental data, and the supplementary numerical data in Table S2 of the Appendix summarise the values of the model parameters for all microorganisms and viscoelastic models under study. As can be seen in Fig. 7 and Table S2 of the Appendix, different organisms have different growth rates. *E. coli* has the highest growth rate followed by *L. monocytogenes* and *P. aeruginosa* (which have similar growth rates), while *L. lactis* has the slowest growth rate for all viscoelastic models under study. Our kinetic trends are similar to the ones reported in literature for those microorganisms in other 3D systems^7,14,71^.

When looking at the macroscopic growth evolution of a specific microorganism on the surface of the developed viscoelastic models of different fat concentrations, no significant differences were observed in the microbial kinetics with increasing fat content and this trend was consistent for all microorganisms under study (Fig. 7, Table S2 of the Appendix). There are very limited studies in literature reporting the microbial dynamics of those microorganisms under changing fat concentrations, and those studies, as mentioned previously, were conducted in very different systems (real food or models) and/or temperatures^20,72,74,104–107^. For example, Baka et al*.* studied *L. monocytogenes* in Frankfurter sausages with increasing fat (0–26%) and showed a reduction on the microbial growth rate for higher fat concentrations at all temperatures under study (4, 8, 12 °C)^108^. Hauerlandová et al. studied the kinetics of spore forming bacteria in cheeses of different fat concentrations, i.e., 30%, 40% and 50% (w/w), at 50 °C and observed only a marginal impact of the highest fat concentration on the microbial kinetics and only after long term incubation i.e., more than 28 days^106^. Karina et al. studied the impact of high fat concentrations (20%, 30% and 50%) in a meat emulsion model at 10–30 °C and, similarly to our findings, observed no effect of the fat concentration on *L. monocytogenes* kinetics^105^. Similarly, Verheyen et al. reported no significant differences in the macroscopic growth kinetics of *L. monocytogenes* on the surface of tri-phasic fish based models at low fat concentrations (between 0 and 1%)^14^. However, Verheyen et al. reported in a later study, differences in the maximum population density (µ_max_) in tri-phasic model systems with higher fat concentration, i.e., 5% to 20%^107^.

We also previously reported similar trends to our current study, for surface growth of *Listeria* on the surface of mono-phasic and bi-phasic Xanthan Gum and Xanthan Gum/Whey Protein based 3D viscoelastic models, but for varying Xanthan Gum concentrations^7^. In particular, despite the differences in stiffness for different Xanthan Gum concentrations, no differences were observed on the microbial growth dynamics. Similarly, to those findings, looking at the rheological properties of the currently developed fat rich systems, as per Fig. 2 and Table S1 of the Appendix, despite the increased gel firmness/stiffness when increasing the fat concentration, no differences are reflected in the microbial kinetics, indicating that the surface stiffness had no effect on the microbial growth. There are studies reporting a reduced microbial growth for increased concentrations of other gelling agents namely gelatine, i.e., in concentration range 1–35% (w/v)^16,17,67,109–115^ and agar, i.e., in concentration range 1–10% (w/v)^116–120^. However, both agar and gelatine differ substantially in terms of their rheological and structural properties as compared to Xanthan Gum, and such differences in stiffness could lead to a completely different microbial behaviour^7^.

#### Microscopic analysis of microbial spatial distribution on the tri-phasic 3D models

**Figure 8:**
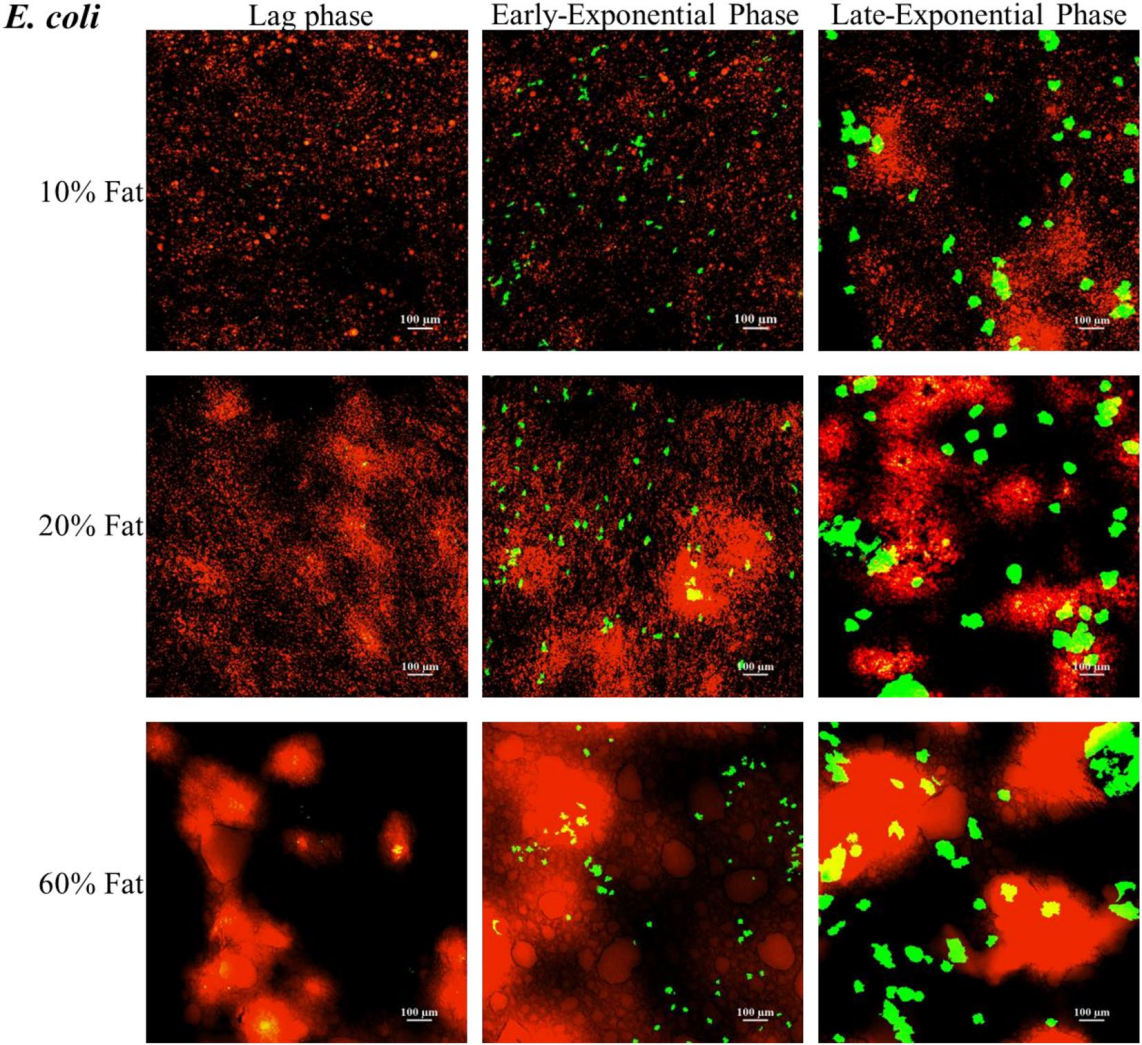
CLSM images of the spatial distribution of *E. coli* (green fluorescence) on the surface of the developed tri-phasic 3D viscoelastic models. Images were taken at different stages of growth, namely 3 h (lag phase), 5 h (mid-exponential phase) and 7 h (late-exponential phase) of incubation at 37 °C. The fat phase is stained with NileRed (red fluorescence).

**Figure 9:**
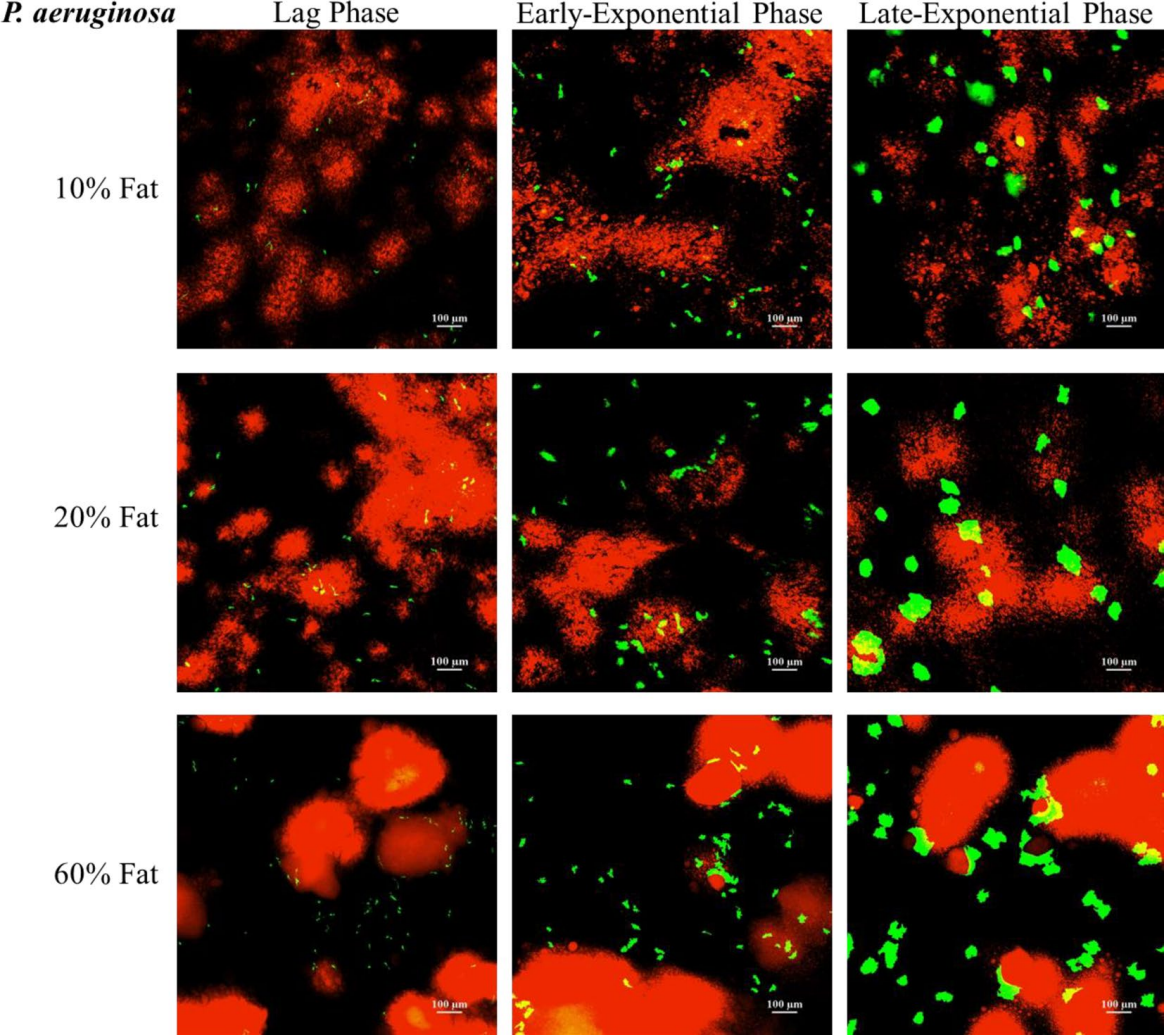
CLSM images of the spatial distribution of *P. aeruginosa* (green fluorescence) on the surface of the developed tri-phasic 3D viscoelastic models. Images were taken at different stages of growth, namely 3 h (early-stationary phase), 5 h (mid-exponential phase) and 7 h (late exponential phase) of incubation at 37 °C. The fat phase is stained with NileRed (red fluorescence).

In order to obtain an understanding of the spatial organisation/distribution of bacteria on the surfaces of the developed 3D models, selective staining of the fat phase was performed followed by CLSM imaging. Figures 8 and 9, show the spatial distribution of *E. coli* and *P. aeruginosa* respectively, on all developed viscoelastic models, at different stages of growth, i.e., lag phase (3 h), mid- exponential phase (5 h) and late-exponential phase (7 h).

As can be seen on Figs. 8 and 9, in contrast to the macroscopic kinetics which were unaffected by the fat concentration (Fig. 7), there are substantial differences in the size of the bacterial colonies for different fat concentrations, especially at 5 and 7 h of growth. The bacterial colonies are generally bigger in size for increasing fat concentration for both *E. coli* (Fig. 8) and *P. aeruginosa* (Fig. 9). A similar trend was observed from the images obtained for *L. lactis* (data not shown). The similarity of the trends points that the impact of the fat concentration on the colony size/formation of all bacteria under study on the models is not strain nor cell wall (gram^+/−^) dependent.

*To the best of our knowledge this is the first systematic study showing the spatiotemporal distribution/mapping of the aggregates/colonies of different foodborne bacteria on the surface of complex (multi-phase) models of increasing fat concentration*.

The differences we see can be attributed to the stiffness differences of the models, i.e., higher fat concentration has resulted in stiffer gels (see Appendix, Table S1). Similarly, we have previously reported an increase in the colony size of *Listeria* on mono-phasic Xanthan Gum gels for increasing concentration of the Xanthan Gum, the latter leading to stiffer gels^7^. In terms of fat-rich systems, Verheyen et al. studied the growth of *L. monocytogenes* in a structured fish-based model system consisting of 1%, 5%, 10% and 20% (v/v) fat and observed bigger colony formation for *L. monocytogenes* for 10% and 20% fat at day 14 of incubation at 10 °C^72^.

**Figure 10:**
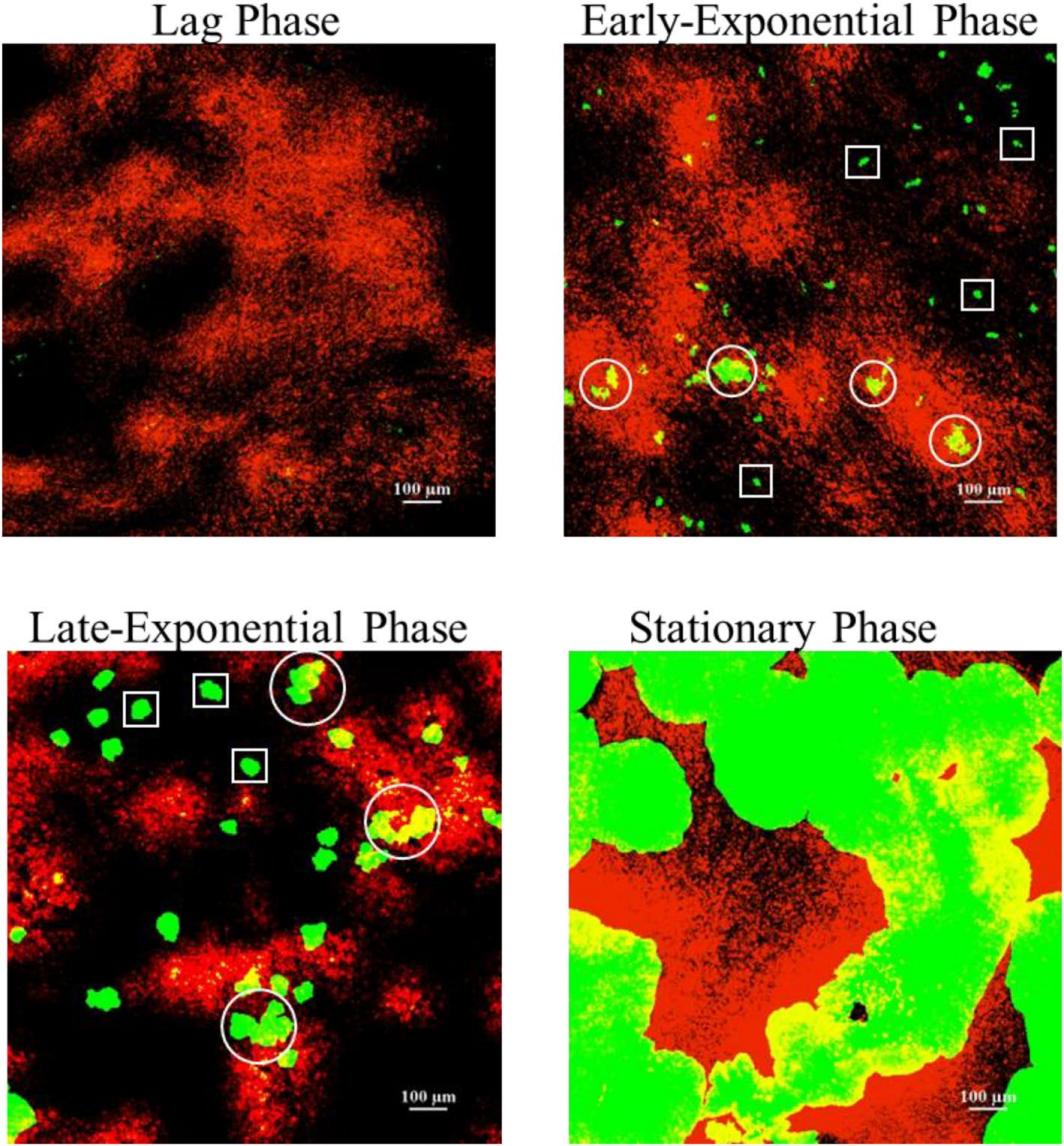
Representative CLSM highlighting the differences in growth of *E.coli* (green fluorescence) on the 20% fat tri-phasic 3D viscoelastic model surface at all growth stages, i.e., lag phase (3 h), mid- (5 h) and late-exponential (7 h), as well as at (late) stationary phase (24 h). Colonies located in fat rich environments are annotated with circles and colonies located on a fat-poor environment are annotated with squares. Fat is stained with NileRed (red fluorescence).

With respect to the spatial distribution of the size of the colonies on the surface of a specific 3D model, as can be seen in Figs. 8, 9 and 10, colonies grown in close proximity to fat are significantly larger and/or cluster more as compared to colonies grown less close to fat- rich areas of the models. This trend is more evident at 5 h and 7 h. However, when reaching stationary phase, due to space limitations, as expected, there is an overgrowth and merging of individual bacterial colonies, which consequently cover all phases of the model (Fig. 10).

In terms of the microbial growth location within the models, as shown in Fig. 10, the bacteria appear to grow primarily at the interface between the fat droplets. This is particularly evident at 5 h (mid-exponential phase) and 7 h (late-exponential phase) of growth. In particular, bacterial growth occurs preferentially on the surface of the protein/polysaccharide phase of the 3D model, but in close proximity to the fat. Only minimal overlapping/overgrowing of the bacterial colony with the fat phase is observed (Fig. 10). Such growth preference/selectivity is less obvious later (Fig. 10), due to outgrowth of colonies and space limitations, as previously mentioned. The preferential growth of bacteria at the protein/fat interface has been previously reported for dairy products, as summarised by Hickey et al.^102^. Laloy et al. was the first study to report the preferential growth of bacteria (starter culture) near the fat phase of cheddar cheese products^121^. There are very few studies discussing the growth location/spatial distribution of micro-organisms in complex model systems, especially as there are very limited studies in complex multi-phase 3D models. Boons et al*.* studied the location of *E. coli* in a gelatin/dextran 3D gel system and showed preferred growth in the polysaccharide (dextran) phase^17^. We have reported selective growth of *L. innocua* and *L. lactis* on the protein phase of our bi-phasic viscoelastic 3D Xanthan Gum/Whey Protein system^7,8^. Furthermore, Verheyen et al. (2019) investigated the effects of fat concentrations (1%, 5%, 10% and 20%) on *L. monocytogenes* when grown in different multi-phase fish-based model systems (liquid, xanthan (high-viscosity liquid), aqueous gel, and emulsion and gelled emulsion systems). Similarly to our observations in our models, this study also reported that the colony size increased in the higher fat concentrations of 10% and 20%, and also reported higher microbial growth affinity towards the fat/water interface^72^.

Overall, the multilevel (macroscopic kinetics vs microscopic imaging) analysis of the microbial behaviour on the novel tri-phasic viscoelastic models reveal discrepancies between the bulk microbial dynamics and the colony level behaviour (colony size distribution and growth location). Whilst no differences were captured in the microbial dynamics, substantial differences were observed in the size of the colonies for different fat concentrations (larger colonies for higher fat) as well as in their growth location, especially during mid- and late- exponential growth phases, i.e., a growth phase which could realistically occur in case of contamination in industry. Spatial growth becomes particularly important in actual food products where, as discussed above and shown with the microscopy analysis in the present study, high structural, biochemical, and spatial complexity occurs. Such microscopic variations in terms of colony size and growth location could affect the efficiency of novel processing technologies, jeopardizing food safety^8,9,12,21,122–124^. For example, larger colonies would be generally more susceptible to surface and/or antimicrobial treatments, i.e., a larger number of cells or more surface area of the colony would be exposed to a surface treatment like plasma or to an antimicrobial compound applied on the surface of a product. Furthermore, inter-species interactions can be hugely affected^8^. Therefore, for an accurate prediction of the microbial response to treatments, it is of paramount importance to couple bulk kinetics with microscopic analysis and to also select representative models to design and validate food safety approaches.

## Conclusion

In this study we have developed and fully characterised novel tri-phasic viscoelastic 3D in vitro models, for microbiological studies. Specifically, we have enriched our previously developed polysaccharide (Xanthan Gum)/ protein (Whey Protein) model with a fat phase (Sunflower Oil) at various concentrations, i.e., 10%, 20%, 40% and 60% (v/v), for better mimicry of real food products. A thorough textural and physicochemical analysis of the models revealed their similarity to various food products. Furthermore, selective staining of the different phases of the models and advanced microscopy imaging of the developed models and of various actual food products, revealed the similarity of the models to food in terms of the spatial distribution and size of the fat droplets, especially cheese products. Microbial experiments of foodborne bacteria, i.e., *L. monocytogenes*, *E. coli*, *P. aeruginosa* and *L. lactis* have revealed very interesting results regarding growth and distribution of cells at colony level. The size of the colonies formed on the surface of the models increased substantially for increasing fat concentrations, especially in mid- and late- exponential growth phases. Furthermore, colonies formed in proximity to fat were substantially larger as compared to the ones that were located far from the fat. In terms of growth location, the majority of colonies were located on the protein/polysaccharide areas rather than the fat areas. All those differences at microscopic level were not captured with the bulk macroscopic kinetics, which were unaffected from changes in fat concentration. This points out the importance of conducting such multilevel analysis as well as the importance of correlating the bacterial spatial growth to the structural characteristics of the system. Even small biochemical or structural differences have been shown to impact the cell distribution and colony size on the surface of the 3D models. This could directly affect the cell response to surface antimicrobial treatments, potentially jeopardising their efficiency. Future work should evaluate additional temperatures, e.g. storage temperatures, to validate the observed trends in temperature conditions that can occur throughout the food production and supply chain, as well as evaluate immersed microbial growth (within/throughout) the model system. Another factor to be considered is the ratio and/or type of components in the model (e.g. the polysaccharides and protein levels or sources) as well as the homogenisation approach as these could affect the structure of the model as well as the distribution of its’ components, consequently affecting the microbial evolution. Finally, evaluation of co-cultures could lead to better understanding of the interaction of pathogens with intrinsic bacteria.

### Supplementary Information

Supplementary Information.
