# Supplementary material for: Unravelling the impact of fat content on the microbial dynamics and spatial distribution of foodborne bacteria in tri-phasic viscoelastic 3D models
Source: Sci Rep. 2023 Dec 9;13:21811. doi: 10.1038/s41598-023-48968-8 (PMC10710490; doi:10.1038/s41598-023-48968-8)
Supplement: Supplementary file 1 — Supplementary Information. [file 41598_2023_48968_MOESM1_ESM.docx]

# Appendix

## (A) Supplementing Data ‘Rheological characterisation’

**Table S1:** Rheological parameters for the characterisation of viscoelastic food models. Mean values (± SD) for storage modulus (Pa) G’, loss modulus (Pa) G’’, and loss tangent (G’/G’’) tanδ, for 0%, 10%, 20%, 40% and 60% fat systems at 7 °C, 25 °C, and 37 °C.

|  |  |  |  |  |  |  |  |  |  |  |
| --- | --- | --- | --- | --- | --- | --- | --- | --- | --- | --- |
|  |  |  |  |  |  |  |  |  |  |  |
| **Viscoelastic 3D models (fat concentration)** | **Temper- ature** | **Storage modulus (Pa) G'** | | | **Loss modulus (Pa) G''** | | | **Loss tangens (G'/G'') Tanδ** | | |
|  |  |  |  |  |  |  |  |  |  |  |
|  |  |  |  |  |  |  |  |  |  |  |
| **0%** | 7 °C | 2386.81 | ± | 93.388 | 510.444 | ± | 16.184 | 0.219 | ± | 0.004 |
|  | 25 °C | 1796.987 | ± | 78.001 | 369.842 | ± | 13.255 | 0.211 | ± | 0.004 |
|  | 37 °C | 1961.176 | ± | 84.126 | 398.68 | ± | 12.584 | 0.210 | ± | 0.005 |
| **10%** | 7 °C | 2454.686 | ± | 97.304 | 485.772 | ± | 15.616 | 0.202 | ± | 0.004 |
|  | 25 °C | 2080.121 | ± | 76.149 | 403.197 | ± | 10.713 | 0.198 | ± | 0.004 |
|  | 37 °C | 2159.229 | ± | 79.277 | 407.229 | ± | 10.937 | 0.194 | ± | 0.004 |
| **20%** | 7 °C | 2662.496 | ± | 95.552 | 534.075 | ± | 10.005 | 0.206 | ± | 0.005 |
|  | 25 °C | 2091.902 | ± | 79.514 | 397.012 | ± | 11.792 | 0.194 | ± | 0.004 |
|  | 37 °C | 2477.332 | ± | 89.916 | 466.743 | ± | 11.431 | 0.194 | ± | 0.005 |
| **40%** | 7 °C | 3671.503 | ± | 119.478 | 635.549 | ± | 19.591 | 0.175 | ± | 0.003 |
|  | 25 °C | 3124.756 | ± | 95.976 | 511.019 | ± | 13.413 | 0.166 | ± | 0.003 |
|  | 37 °C | 3593.215 | ± | 138.583 | 608.216 | ± | 19.083 | 0.174 | ± | 0.005 |
| **60%** | 7 °C | 5113.658 | ± | 249.879 | 788.722 | ± | 41.518 | 0.155 | ± | 0.003 |
|  | 25 °C | 5190.529 | ± | 200.253 | 719.291 | ± | 29.018 | 0.140 | ± | 0.003 |
|  | 37 °C | 5361.499 | ± | 216.15 | 773.214 | ± | 27.921 | 0.148 | ± | 0.005 |
|  |  |  |  |  |  |  |  |  |  |  |
|  |  |  |  |  |  |  |  |  |  |  |

## (B) Supplementing Data ‘Structural microscopic analysis’


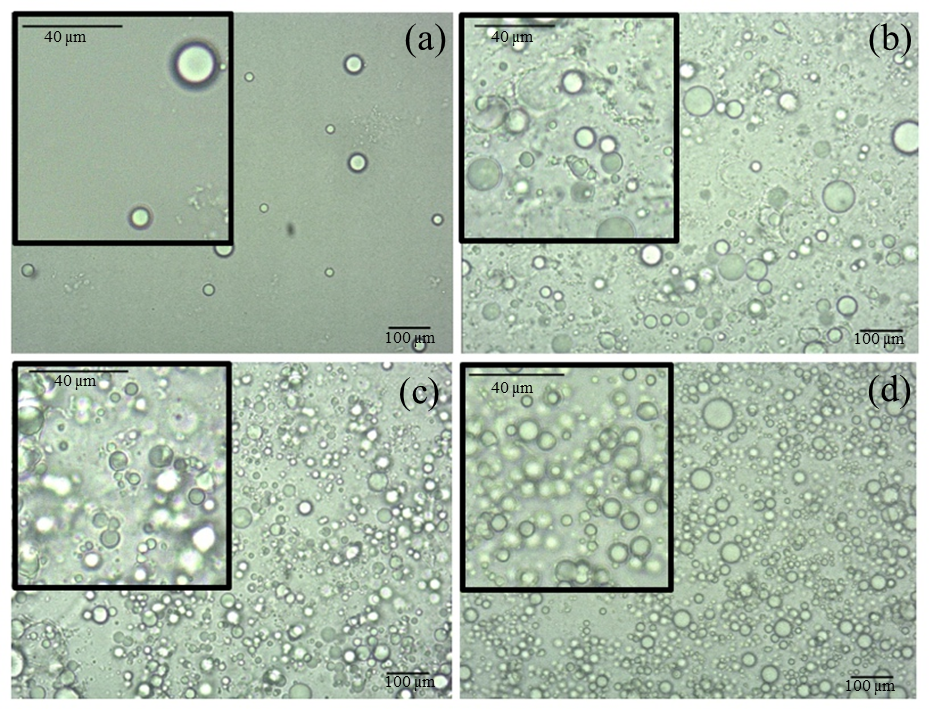
 **Figure S1:** Light microscopy image of the fat globules of the food model systems with increasing fat concentration of (a) 10%, (b) 20%, (c) 40% and (d) 60%. The images show 10x magnifications and 40x zoom images respectively.

## (C) Supplementing data ‘ Macroscopic surface growth department’

**Table S2:** Baranyi & Roberts growth kinetic parameters for the surface growth of *L. monocytogenes*, *E. coli*, *P. aeruginosa* and *L. lactis* on all tri-phasic viscoelastic models under study.

|  |  |  |  |  |  |
| --- | --- | --- | --- | --- | --- |
|  |  |  |  |  |  |
| **Model Parameters** |  | **0% fat concentration** | |  |  |
|  |  | ***L. monocytogenes*** | ***E. coli*** | ***P. aeruginosa*** | ***L. lactis*** |
|  |  |  |  |  |  |
|  |  |  |  |  |  |
| N_max_ (lnCFU/mL) | | 18.2541 | 19.5396 | 19.3455 | 18.1039 |
| SD (N_max_) |  | 0.3660 | 0.4094 | 0.4988 | 0.4154 |
| µ_max_ (1/h) |  | 1.5931 | 1.9715 | 1.5829 | 1.0085 |
| SD (µ_max_) |  | 0.1561 | 0.2324 | 0.1879 | 0.0788 |
| λ (h) |  | 2.7447 | 1.6456 | 1.3897 | 2.4012 |
| SD (λ) |  | 0.5198 | 0.5996 | 0.7950 | 0.6413 |
|  |  |  |  |  |  |
|  |  |  |  |  |  |
|  |  | **20% fat concentration** | |  |  |
|  |  | ***L. monocytogenes*** | ***E. coli*** | ***P. aeruginosa*** | ***L. lactis*** |
|  |  |  |  |  |  |
|  |  |  |  |  |  |
| N_max_ (lnCFU/mL) | | 18.5145 | 19.5933 | 19.4244 | 17.7243 |
| SD (N_max_) |  | 0.3473 | 0.3808 | 0.4665 | 0.5166 |
| µ_max_ (1/h) |  | 1.5517 | 2.0135 | 1.5069 | 1.0527 |
| SD (µ_max_) |  | 0.1244 | 0.2225 | 0.1642 | 0.1186 |
| λ (h) |  | 2.6086 | 1.7821 | 1.1856 | 2.7152 |
| SD (λ) |  | 0.4470 | 0.5462 | 0.7809 | 0.8250 |
|  |  |  |  |  |  |
|  |  |  |  |  |  |
|  |  | **60% fat concentration** | |  |  |
|  |  | ***L. monocytogenes*** | ***E. coli*** | ***P. aeruginosa*** | ***L. lactis*** |
|  |  |  |  |  |  |
|  |  |  |  |  |  |
| N_max_ (lnCFU/mL) | | 18.4010 | 19.4116 | 19.3681 | 17.0951 |
| SD (N_max_) |  | 0.3559 | 0.3556 | 0.4220 | 0.4144 |
| µ_max_ (1/h) |  | 1.5435 | 1.9561 | 1.5116 | 1.1430 |
| SD (µ_max_) |  | 0.1424 | 0.2037 | 0.1534 | 0.1328 |
| λ (h) |  | 2.4480 | 1.6998 | 0.9872 | 2.4772 |
| SD (λ) |  | 0.5280 | 0.5235 | 0.7276 | 0.7540 |
|  |  |  |  |  |  |
|  |  |  |  |  |  |
